# Positive and Negative Photoconductivity Conversion Induced by H_2_O Molecule Adsorption in WO_3_ Nanowire

**DOI:** 10.1186/s11671-019-2978-y

**Published:** 2019-04-24

**Authors:** Yahui Liu, Peng Fu, Yanling Yin, Yuehua Peng, Wenjun Yang, Gang Zhao, Weike Wang, Weichang Zhou, Dongsheng Tang

**Affiliations:** Synergetic Innovation Center for Quantum Effects and Application, Key Laboratory of Low-dimensional Quantum Structures and Quantum Control of Ministry of Education, School of Physics and Electronics, Hunan Normal University, Changsha, 410081 People’s Republic of China

**Keywords:** Hexagonal WO_3_ nanowire, Negative photoconductivity, Positive photoconductivity, Hydrogen ion, Schottky emission

## Abstract

Negative photoconductivity effect has been observed in the Au/WO_3_ nanowire/Au devices in a high humidity environment, which might be attributed to the accumulation of H^+^ ions on the surface of WO_3_ nanowire. Under illumination with violet light (445 nm), the photo-excited holes can oxidize the adsorbed H_2_O molecules to produce H^+^ ions and O_2_, while the photo-excited electrons at the conduction band bottom do not have enough energy to reduce H^+^ ions. These H^+^ ions will accumulate on the surface of the hexagonalWO_3_ nanowire. They will capture mobile electrons and then reduce the concentration of carriers, which will result in a significant increase in the height of interface barrier and then a significant decrease in the conductance of the Au/h-WO_3_ nanowire/Au device. By adjusting the relative humidity, light intensity, or bias voltage, the concentration and distribution of H^+^ ions and then the conversion between positive and negative photoconductivity, as well as resistive switching properties, can be well regulated in this kind of devices.

## Introduction

Tungsten oxide (WO_3_) exhibits excellent photo-(electro-, gas-, thermo-)chromic properties and resistive switching behaviors [1–13], which might be attributed to its distinctive crystal and electronic band structures. WO_3_ is constructed from WO_6_ octahedra by sharing the equatorial oxygen atoms, which leaves more empty interstices in the oxygen sub-lattice. As a result, WO_3_ can accommodate external species such as hydrogen ions and alkali metal ions into its solid framework to form stable nonstoichiometric intercalation compounds with the color from yellowish green to gold and the conductivity from insulator to metal. Meanwhile, the bottom of the conduction band of WO_3_ lies below the hydrogen ion reduction level, while the top of the valence band lies above the level of H_2_O molecular oxidation. Therefore, H_2_O molecules adsorbed on the surface of WO_3_ can be oxidized to produce hydrogen ions (H^+^ ions) and O_2_ by the excited or injected holes at the top of the valence band, while H^+^ ions cannot be reduced by electrons at the bottom of the conduction band. Generally speaking, the coloring or resistive switching of WO_3_ in atmospheric environment under external excitation such as illumination and bias voltage can be attributed to the H^+^ ions embedded in the lattice [14, 15].

Therefore, it is possible to manipulate the optical and resistive switching properties of WO_3_ by regulating the transport and distribution of H^+^ ions in the lattice or on the surface of WO_3_. Single crystalline hexagonal WO_3_ nanowire (h-WO_3_ NW) possessing large specific surface area and conductive channel might be an ideal platform for studying the effect of the H^+^ ions produced by H_2_O oxidation. In our previous work, the single-crystalline h-WO_3_ NWs grown along the *c* direction do exhibit memristive effect or resistive switching phenomenon, which can be enhanced remarkably and even regulated by the H^+^ ions produced by the oxidation of the adsorbed H_2_O molecules [16–19].

In this letter, we explored the photoconductivity of h-WO_3_ NW under different relative humidity and found that the positive photoconductivity (PPC) effect is always accompanied by negative photoconductivity (NPC) effect in a high relative humidity environment. By adjusting the relative humidity, light intensity, or bias voltage, it is possible to manipulate the creation, distribution, and annihilation of H^+^ ion son the surface of WO_3_ and then regulate the concentration of carriers in the WO_3_ nanowire and the height of the interface barrier.

## Methods

### WO_3_ Nanowire Synthesis

The h-WO_3_ nanowires used in this investigation were synthesized using a simple hydrothermal method as previously reported [20, 21]. In a typical synthesis, 8.25 g sodium tungstate (Na_2_WO_4_·2H_2_O) was dissolved in 250 mL deionized water. Hydrochloric acid (HCl, 3 M) was used to adjust the PH value of the Na_2_WO_4_ solution to 1.2. After being filtered, the precipitate was washed sequentially with deionized water and ethanol to remove contaminant ions and then dispersed in 200 mL citric acid (C_6_H_8_O_7_, 0.1 M) to form a translucent homogeneous and stable WO_3_ sol. A 45-mL volume of WO_3_ sol was transferred into a 50-mL autoclave, and then 1.3 g potassium sulfate (K_2_SO_4_,) was added to the sol. The autoclave was sealed and maintained at 240 °C for 32 h, and then cooled down to room temperature. The precipitates in the solution were filtered, washed sequentially with deionized water and ethanol to remove possible remnant ions, and then dried at 60 °C.

### Device Fabrication

The individual h-WO_3_ nanowire-based devices were fabricated on heavily n-doped Si substrate covered with a 100 nm thick thermally grown SiO_2_ layer. Electrodes were defined on the Si substrate with WO_3_ nanowires by using a standard photo-lithography technique (ABM, Inc., San Jose, CA (405)) and formed by metal deposition (100-nm-thick Au) and a lift-off process.

### Electrical Measurement

Electrical transport measurements were conducted on a probe station at room temperature by using semiconductor characterization systems (Keithley 2602). The probe station is placed in a homemade vacuum chamber, which is firstly vacuumized to a base pressure less than 10^−1^ Pa by a mechanical pump. The relative humidity (RH) in the environment was adjusted by evaporation of deionized H_2_O and a dehumidifier. The accuracy of the humidity sensor used in our experiments was about ± 1%.

## Results and Discussion

**Fig. 1:**
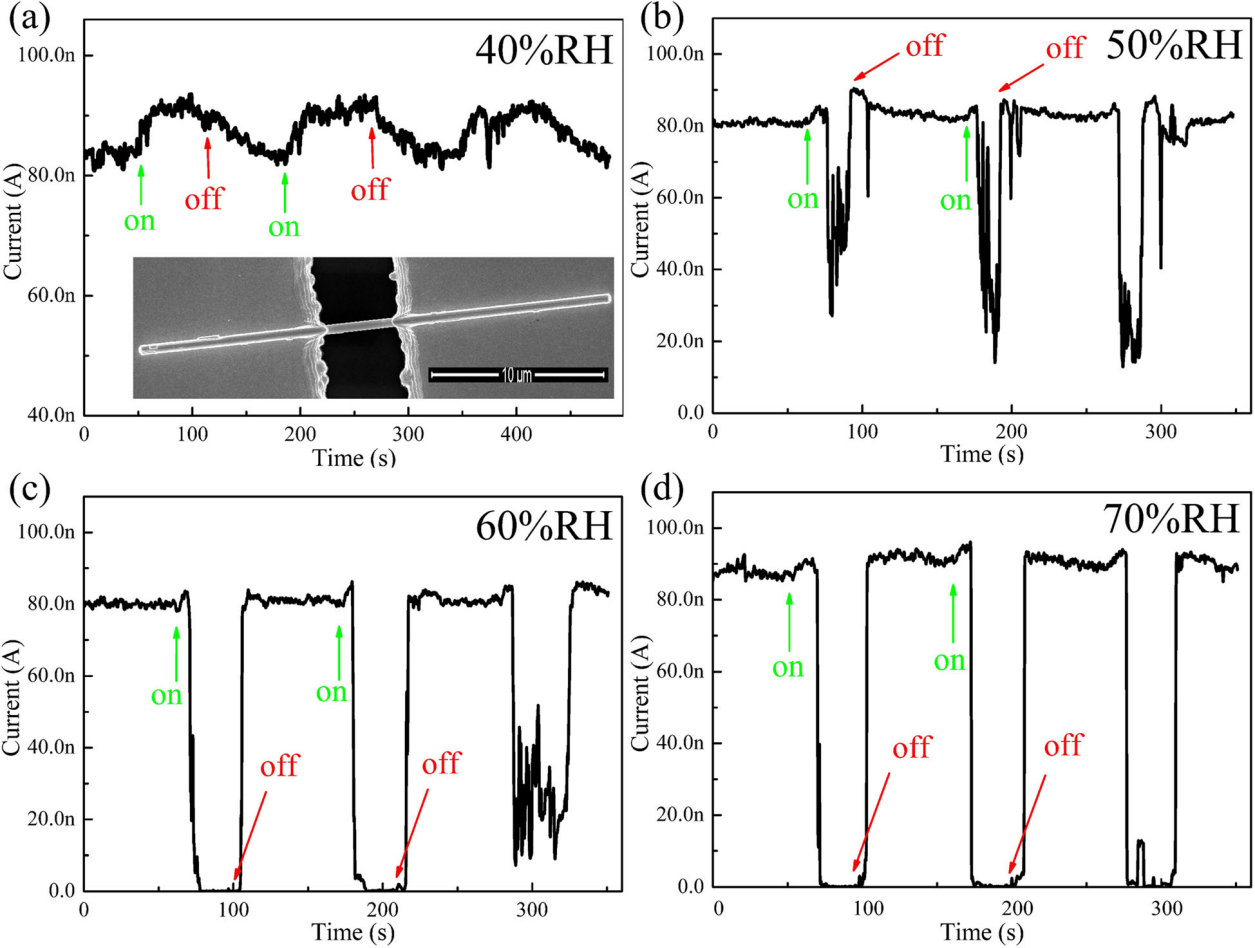
Typical *I-T* curves (*V*_ds_ = 3 V) of an Au/h-WO_3_ NW/Au device recorded repeatedly with laser (445 nm, 500 mW) on and off under 40%RH (**a**), 50% RH (**b**), 60% RH (**c**), and 70% RH (**d**). The lower inset of **a**: SEM image of an Au/h-WO_3_ NW/Au device, the nanowire between two electrodes with a diameter of about 300 nm, and a length of about 4 μm

Figure 1 shows the typical current-time (*I-T*) curves of an Au/h-WO_3_ NW/Au device recorded with laser (445 nm, 500 mW) on and off under different RH levels. When the RH is 40% (Fig. 1a), the current rises slightly under illumination, which is the normal PPC due to the inter-band transition [22, 23]. As the RH increases to 50% (Fig. 1b), the current rises slightly when the laser is turned on. And then, after about 10 s, the photocurrent drops significantly, namely the intriguing NPC effect. With increasing the RH gradually, the device exhibits the more excellent and stable NPC as shown in Fig. 1c, d. The NPC effect has been reported in some nanomaterials [24–26], but never been observed in WO_3_. Preliminarily, the NPC effect of WO_3_ nanowire might be attributed to the adsorbed H_2_O molecules on the surface. After all, H_2_O molecule adsorption and photo-desorption have been proved to play an important role in determining the photoelectric properties and lead to NPC effect in nanoscale materials [27–29]. It means that the conductance of these nanoscale materials depend sensitively on the amount of adsorbed H_2_O molecules. However, unlike the photocurrents, the dark currents recorded under the different RH levels are almost the same (80 nA) as shown in the Fig. 1, which proves that the changes in the photocurrents under different RH levels cannot simply be attributed to photo-induced desorption H_2_O molecules. Therefore, there is a new physical mechanism answering for the NPC effect of the h-WO_3_ NW. In addition, the dark current in Fig. 1d is slightly larger than 80 nA. When the RH is very high, more H_2_O molecules are adsorbed on the WO_3_ NW and can form the H_2_O film on the surface of WO_3_. And this layer of water molecule can increase the conductance of the device based on the Grotthuss mechanism [30]. Therefore, the dark current in Fig. 1d increases slightly.

**Fig. 2:**
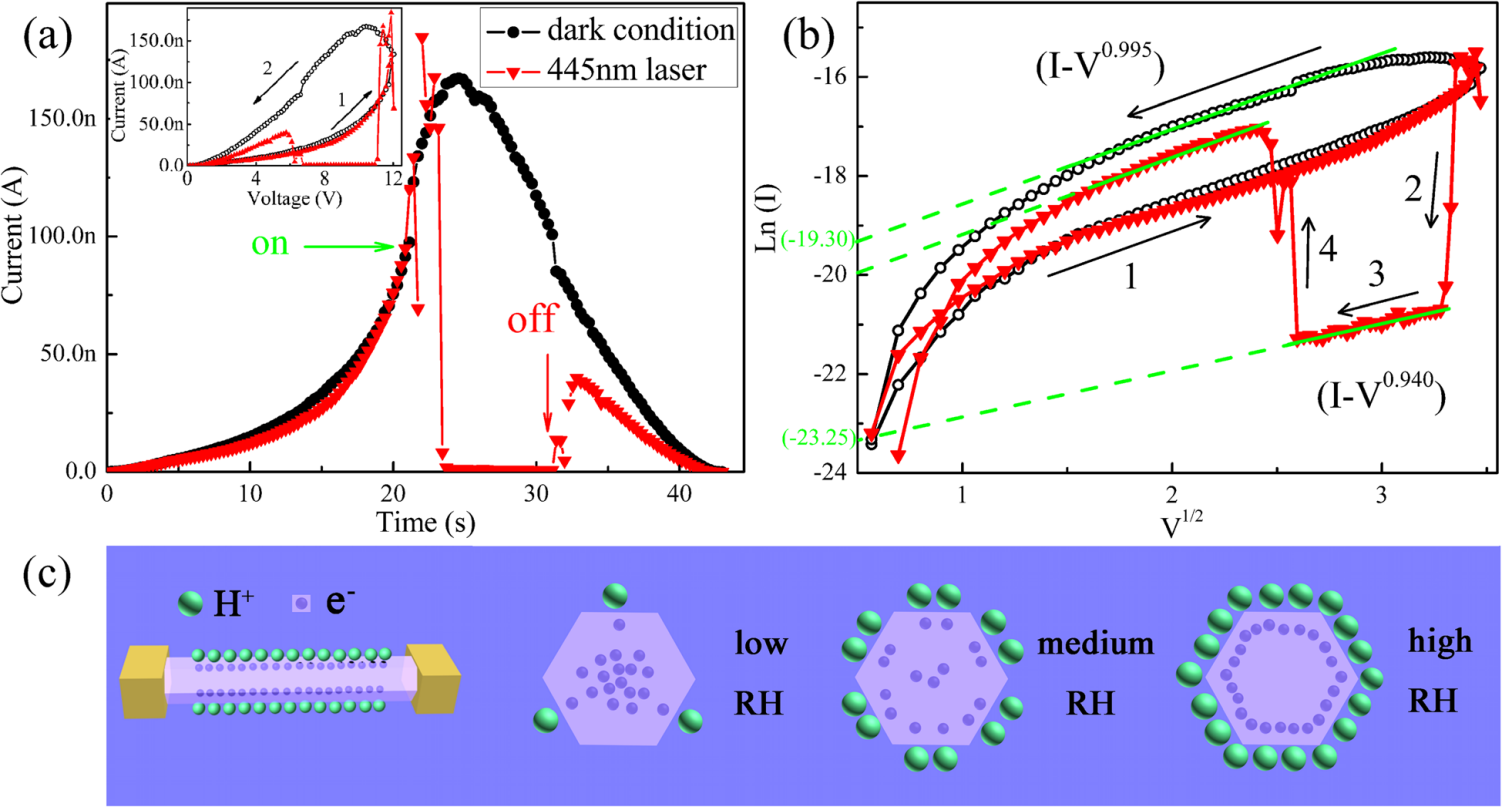
**a** *I-T* curves recorded on a bias sweep range of 12 V in the dark and under illumination (445 nm, 500 mW) at 70% RH. **b** The plots of *ln(I)* versus *V*^*1/2*^. **c** Diagram of the mechanism of the NPC of the WO_3_ NW. The inset of **a**: the *I-V* curves on a bias sweep range of 12 V

To elucidate the origins of the NPC, the involving conductive mechanism needs to be determined firstly. As shown in the inset of Fig. 2a, the typical current-voltage (*I-V*) curve is recorded with the bias voltage scanning and the laser on and off under the 70% RH, which indicates NPC effect as well as resistive switching. For the purpose of making an obvious contrast, the *I-V* curves were converted to the *I-T* curves as displayed in Fig. 2a and replotted based on the Schottky law (*lnI*∝*V*^*1/2*^) [31]. For both photocurrent and dark current, *lnI* is linear with *V*^*1/2*^ under high bias voltage. The conduction mechanisms for both cases are Schottky emission and barrier height which can be obtained from the intercept of the Schottky plot. The Schottky barrier under light illumination is much higher than that in dark environment as indicated by the green intercepts in Fig. 2b. Therefore, the NPC effect of the h-WO_3_ NW might be attributed to the increase of the Schottky barrier height induced by violet light illumination. As previously reported [15], the resistive switching properties this kind of devices has can be enhanced remarkably by adsorbed H_2_O molecules. In that situation, the holes injected from the positively charged electrode oxidize the adsorbed H_2_O molecules producing H^+^ ions and O_2_, while the electrons injected from the negatively charged electrode under small bias voltage do not have enough energy to reduce H^+^ ions because of the peculiar electronic band structure of WO_3_. The H^+^ ions produced by H_2_O oxidation will accumulate gradually on the surface under continuous bias scanning, which will deplete all mobile electrons in the WO_3_ nanowire. Therefore, under illumination with violet light (445 nm), the photo-excited holes can also oxidize the adsorbed H_2_O molecules to produce H^+^ ions. The only difference is that the H^+^ ions are produced and accumulated faster, which prevents H^+^ ions from entering the lattice of WO_3_ NW more easily to transform it into a metallic state. They will capture mobile electrons to form the electric double-layer and then reduce the concentration of carriers as shown in Fig. 2c, which will result in a significant increase in the height of interface barrier and then a significant decrease in the conductance of the Au/h-WO_3_ NW/Au device. If the RH level is low (less than 50%), there are less than two H_2_O molecular layers on the surface, and the amount of H^+^ ions produced by water oxidation is relatively small. Furthermore, H^+^ ions cannot move freely in the discontinuous layers of H_2_O molecules to accumulate near the negatively charged electrode. Accordingly the ability of localizing mobile electrons is weak or even negligible, and then the device exhibits the PPC effect (Fig. 1a).

**Fig. 3:**
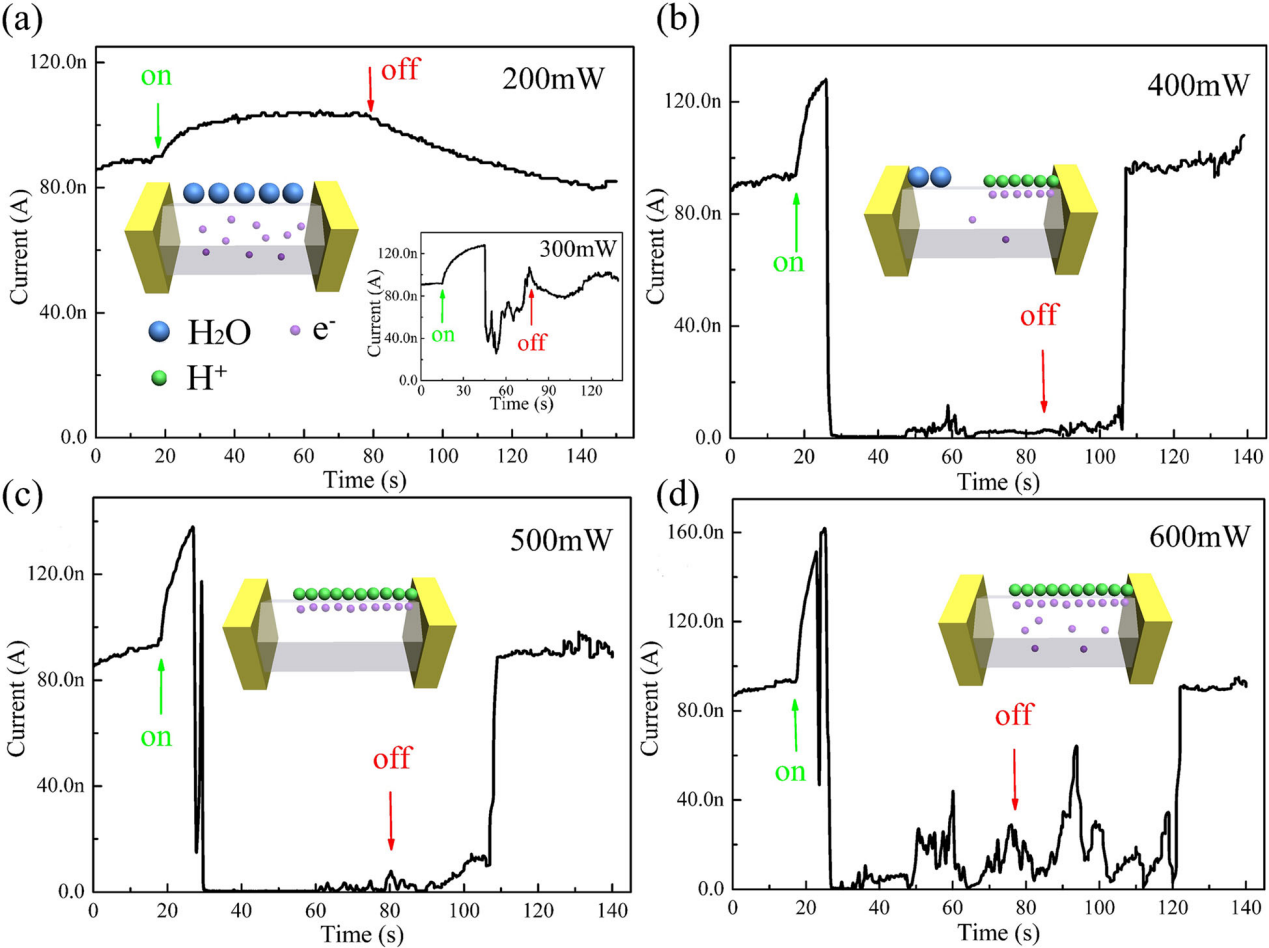
Typical *I-T* curves (*V*_ds_ = 3 V) of an Au/h-WO_3_ NW/Au device recorded repeatedly with laser (445 nm, 200 mW (**a**), 400 mW (**b**), 500 mW (**c**), and 600 mW(**d**)) on and off at 70% RH. The right inset of **a**: the *I-T* curves of 300 mW. The four schematic insets showing the effect of H^+^ ions under different laser powers

To further investigate the origin of the NPC effect and confirm the reasonableness of the above mechanism, the power-dependent *I-T* measurements were carried out systematically as shown in the Fig. 3. When the power of the laser is set at 200 mW, the device exhibits stable PPC effect under illumination (Fig. 3a). As the power increases to 300 mW, some traces of NPC can be clearly observed (the right inset of Fig. 3a). With further increasing laser power from 300 to 400 mW and 500 mW, the current rises quickly at first seconds under illumination exhibiting the PPC effect, and then drops suddenly exhibiting the NPC effect (Fig. 3b, c). Upon switching off the light source, the current does not change significantly until it increases to initial value rapidly after more than 20s. It is clear that the current increases more significantly and drops more quickly with the light intensity increasing, which might be attributed to the rate of hydrogen ion production and aggregation proportional to the light intensity. When the light intensity is weak (less than 200 mW), the efficiency of inter-band transition is very low, and then the generated H^+^ ions are negligible or reduced by hot electrons. When the light intensity is strong, the concentration of carriers (electrons and holes) increases abruptly upon illumination, and then there is the generation and aggregation of hydrogen ions. The conversion from PPC to NPC can be well explained by the process of H^+^ ion accumulation on the surface. When the laser power further increases to 600 mW (Fig. 3d), the photocurrent fluctuates drastically, which might be attributed to the competition between the production and reduction of H^+^ ions. The efficiency of the inter-band transition is so high that the adsorbed H_2_O molecules are consumed fast and cannot be supplied just in time. After all, it takes a certain time for the H_2_O molecules in atmosphere to relax onto the h-WO_3_ NW surface. From the above analysis, we concluded that the productivity of H^+^ ions is dependent on the efficiency of inter-band transition. When the power of laser is low, the efficiency of inter-band transition is relatively low, and it will take more time to produce enough H^+^ ions to achieve the conversion from PPC to NPC effect. In contrast, when the power becomes larger, it will take shorter time to achieve this kind of conversion.

**Fig. 4:**
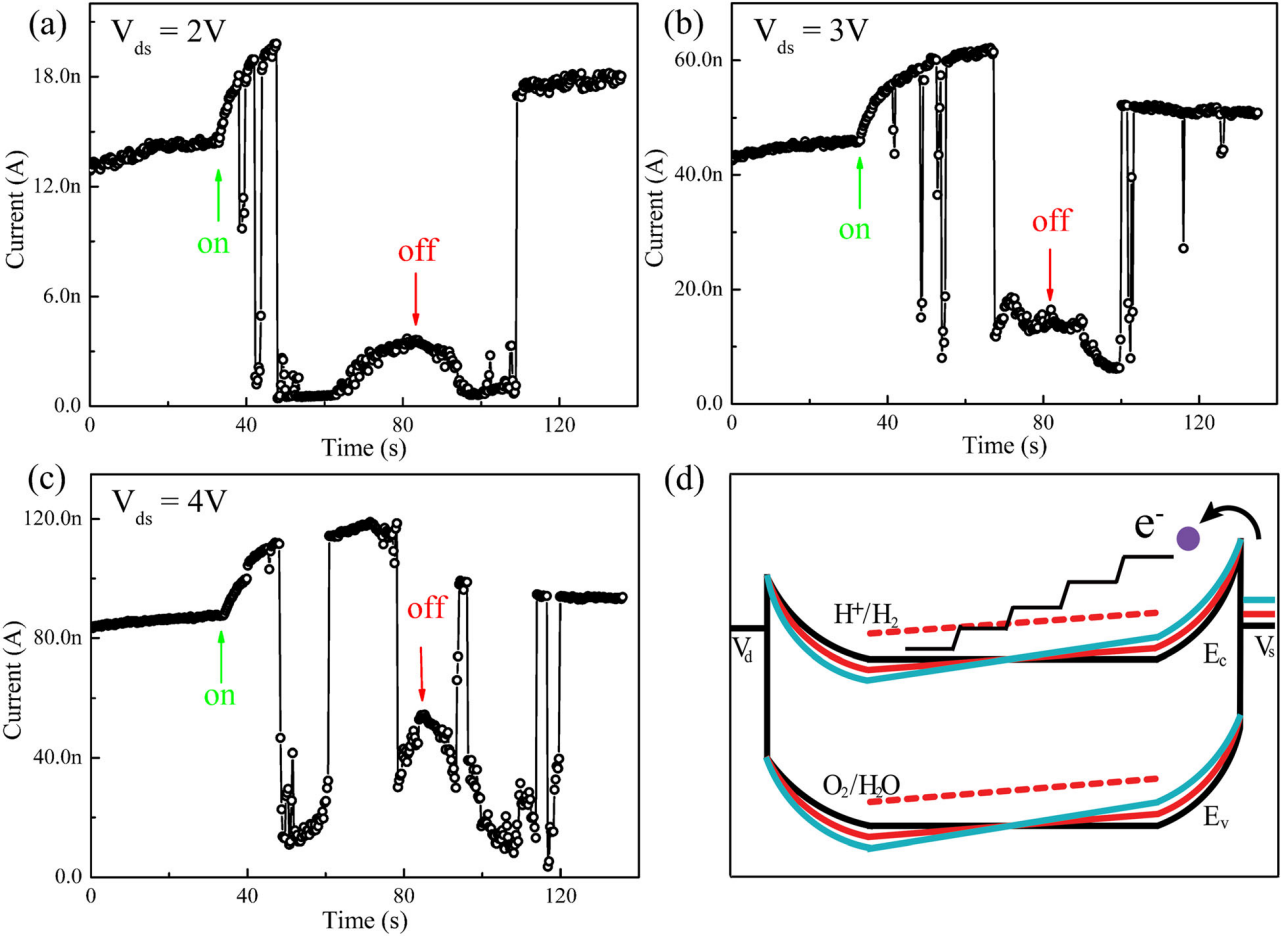
Typical *I-T* curves of a device recorded on different biases (2 V (**a**), 3 V (**b**), 4 V (**c**)) with laser (445 nm, 500 mW) on and off at 50% RH. **d** Schematic band structures of the Au/h-WO_3_ NW/Au device under different bias voltages and the non-ballistic transport of the injected electrons

To further study the regulation of H^+^ ions and then the conversion between the PPC and NPC effect of the h-WO_3_ NWs, the typical *I-T* curves of an Au/h-WO_3_ NW/Au device were measured under different bias voltages as shown in Fig. 4. In this part, the RH level is set at 50%, because the amount of the adsorbed H_2_O molecules is not so much that the effect of the bias voltages might be more obvious. When the bias voltage is 2 V, the NPC in the WO_3_ nanowire is very stable under illumination (445 nm, 500 mW) as shown in Fig. 4a. However, with the bias voltage increasing, the *I-T* curves become more fluctuated as displayed in the Fig. 3b, c). Meanwhile, it also indicates that it takes less time to achieve the conversion from PPC to NPC effect under the small bias voltage. In addition, when the light was switched off, the current decreases a little at first because the photo-excited electrons and holes preferentially recombine as shown in Fig. 4, which is similar to the cases in InN thin film [32] and InAs nanowire [33]. To comprehend this phenomenon completely, the electronic band structure of the Au/h-WO_3_ NW/Au device is shown in the Fig. 4d, which bends gradually with bias voltage increasing. Though the H^+^ ion reduction level lies slightly higher than the bottom of the conduction band of the WO_3_ NW, the number of the hot electrons above the H^+^ ions reduction level injected from the negatively charged electrode based on Schottky emission might be large enough as long as the bias is large enough. These hot electrons exist only near the negatively charged electrode due to their non-ballistic transport behavior and will reduce the accumulated H^+^ ions quickly. As the H^+^ ions disappear, the height of the Schottky barrier decreases, and the voltage dropping on the barrier decreases accordingly. The number of the hot electrons above the H^+^ ion reduction level decreases correspondingly, which will lead to the accumulation of H^+^ ions again. Therefore, for the relatively long h-WO_3_ NW, it is reasonable to consider that the H^+^ ions accumulate and are reduced by hot electrons alternatively, which results in current fluctuating as shown in Fig. 4c.

## Conclusions

In summary, we have systematically investigated the photoelectric properties of the Au/h-WO_3_ NW/Au devices. The experimental results indicate that the h-WO_3_ NW presents excellent and stable NPC effect under high RH, moderate laser power, and small bias voltage. This is because the H^+^ ions produced by H_2_O oxidation on the surface of h-WO_3_ NW will capture mobile electrons and then reduce the concentration of carriers and will result in a significant increase in the height of interface barrier of the Au/h-WO_3_ NW/Au device. By adjusting the relative humidity, light intensity, or bias voltage, the concentration and distribution of H^+^ ions and then the conversion between positive and negative photoconductivity can be well regulated in this kind of devices. This work might help to better understand the behavior of H^+^ ions and offer a new possibility to regulate the optical and resistive switching properties of WO_3_.

## Abbreviations

Au: Aurum
H^+^ ions: Hydrogen ions
h-WO_3_: Hexagonal tungsten trioxide
*I-T*: Current-time
*I-V*: Current-voltage
NPC: Negative photoconductivity
NW: Nanowire
PPC: Positive photoconductivity
RH: Relative humidity

## References

[1] He YP, Wu ZY, Fu ML, Li CR, Miao YM, Cao L (2003). Photochromism and size effect of WO_3_ and WO_3_-TiO_2_ aqueous sol. Chem Mater.

[2] Yamazaki S, Ishida H, Shimizu D, Adachi K (2015). Photochromic properties of tungsten oxide/methylcellulose composite film containing dispersing agents. ACS Appl Mater Inter.

[3] He T, Yao JN (2007). Photochromic materials based on tungsten oxide. J Mater Chem.

[4] Baeck SH, Choi KS, Jaramillo TF, Stucky GD, McFarland EW (2003). Enhancement of photocatalytic and electrochromic properties of electrochemically fabricated mesoporous WO_3_ thin films. Adv Mater.

[5] Kida T, Nishiyama A, Hua ZQ, Suematsu K, Yuasa M, Shimanoe K (2014). WO_3_ nanolamella gas sensor: porosity control using SnO_2_ nanoparticles for enhanced NO_2_ sensing. Langmuir.

[6] Garcia-Sanchez RF, Ahmido T, Casimir D, Baliga S, Misra P (2013). Thermal effects associated with the Raman spectroscopy of WO_3_ gas-sensor materials. J Phys Chem A.

[7] Wang J, Khoo E, Lee PS, Ma J (2008). Synthesis, assembly, and electrochromic properties of uniform crystalline WO_3_nanorods. J Phys Chem C.

[8] Wang J, Khoo E, Lee PS, Ma J (2009). Controlled synthesis of WO_3_ nanorods and their electrochromic properties in H_2_SO_4_ electrolyte. J Phys Chem C.

[9] Santato C, Odziemkowski M, Ulmann M, Auustynski J (2001). Crystallographically oriented mesoporous WO_3_ films: synthesis, characterization, and applications. J Am Chem Soc.

[10] Lee SH, Deshpande R, Parilla PA, Jones KM, To B, Mahan AH (2006). Crystalline WO_3_ nanoparticles for highly improved electrochromic applications. Adv Mater.

[11] Lei L, Yin YL, Liu C, Zhou Y, Peng YH, Zhou F (2017). Resistive switching behavior of hexagonal sodium tungsten bronze nanowire. Solid State Ionics.

[12] Le VQ, Do TH, Retamal JRD, Shao PW, Lai YH, Wu WW, He JH, Chueh YL, Chu YH (2019). Van der Waals heteroepitaxial AZO/NiO/AZO/muscovite (ANA/muscovite) transparent flexible memristor. Nano Energy.

[13] Retamal JRD, Ho CH, Tsai KT, Ke JJ, He JH (2019) Self-organized Al Nanotip electrodes for achieving ultralow-power and error-free memory. IEEE Trans Electron Devices 66:948–943

[14] Cong S, Geng FX, Zhao ZG (2016). Tungsten oxide materials for optoelectronic applications. Adv Mater.

[15] Zhou Y, Peng YH, Yin YL, Zhou F, Liu C, Ling J (2016). Modulating memristive performance of hexagonal WO_3_ nanowire by water-oxidized hydrogen ion implantation. Sci Rep.

[16] He XW, Yin YL, Guo J, Yuan HJ, Peng YH, Zhou Y (2013). Memristive properties of hexagonal WO_3_ nanowires induced by oxygen vacancy migration. Nanoscal Res Lett.

[17] Liu BQ, Tang DS, Zhou Y, Yin YL, Peng YH, Zhou WC (2014). Electrical characterization of H_2_S adsorption on hexagonal WO_3_ nanowire at room temperature. J Appl Phys.

[18] Zhou Y, Yin YL, Peng YH, Zhou WC, Yuan HJ, Qin ZA (2014). Enhanced memristive performance of individual hexagonal tungsten trioxide nanowires by water adsorption based on Grotthuss mechanism. Mater Res Express.

[19] Guo J, Zhou Y, Yuan HJ, Zhao D, Yin YL, Hai K (2013). Reconfigurable resistive switching devices based on individual tungsten trioxide nanowires. AIP Adv.

[20] Gu ZJ, Zhai TY, Gao BF, Sheng XH, Wang YB, Fu HB (2006). Controllable assembly of WO_3_ nanorods/nanowires into hierarchical nanostructures. J Phys Chem B.

[21] Serge Z, Eugene K, Benjamin C, Sivacarendran B (2014). Proton intercalated two-dimensional WO_3_ nano-flakes with enhanced charge-carrier mobility at room temperature. Nanoscale.

[22] Huang K, Zhang Q, Yang F, He DY (2010). Ultraviolet photoconductance of a single hexagonal WO_3_ nanowire. Nano Res.

[23] Ouyang WX, Teng F, He JH, Fang XS (2019). Enhancing the photoelectric performance of photodetectors based on metal oxide semiconductors by charge-carrier engineering. Adv Funct Mater.

[24] Huang YQ, Zhu RJ, Kang N, Du J, Xu HQ (2013). Photoelectrical response of hybrid grapheme-PbS quantum dot devices. Appl Phys Lett.

[25] Tavares MAB, Silva MJ, Peres ML, Castro S, Soares DAW, Okazaki AK (2017). Investigation of negative photoconductivity in p-type Pb_1-x_Sn_x_Te film. Appl Phys Lett.

[26] Lui CH, Frenzel AJ, Pilon DV, Lee YH, Ling X, Akselrod GM (2014). Trion-induced negative photoconductivity in monolayer MoS_2_. Phys Rev Lett.

[27] Peng L, Zhai JL, Wang DJ, Wang P, Zhang Y, Pang S (2008). Anomalous photoconductivity of cobalt-doped zinc oxide nanobelts in air. Chem Phys Lett.

[28] Zhang Q, Jie J, Diao S, Shao Z, Zhang Q, Wang L (2015). Solution-processed grapheme quantum dots deep-UV photodetectors. ACS Nano.

[29] Nakanishi H, Bishop KJM, Kowalczyk B, Nitzan A, Weiss EA, Tretiakov KV (2009). Photoconductance and inverse photoconductance in films of functionalized metal nanoparticles. Nature.

[30] Agmon N (1995). The Grotthuss mechanism. Chem Phys Lett.

[31] Chiu Fu-Chien (2014). A Review on Conduction Mechanisms in Dielectric Films. Advances in Materials Science and Engineering.

[32] Wei PC, Chattopadhyay S, Yang MD, Tong SC, Shen JL, Lu CY (2010). Room-temperature negative photoconductivity in degenerate InN thin films with a super gap excitation. Phys Rev B.

[33] Han YX, Zheng X, Fu MQ, Pan D, Li X, Guo Y (2016). Negative photoconductivity of InAs nanowire. Phys Chem Chem Phys.

